# Antibiotic use in surgical infections at a tertiary teaching hospital in Ghana

**DOI:** 10.4314/gmj.v58i3.5

**Published:** 2024-09

**Authors:** Josephine Mensah, Antoinette A Bediako-Bowan, Amos Amoako-Adusei, Franklin Acheampong, Mohammed Sheriff, Nii A Adu-Aryee

**Affiliations:** 1 Pharmacy Directorate, University of Ghana Medical Centre, Legon, Accra, Ghana; 2 Department of Surgery, University of Ghana Medical School, College of Health Sciences, University of Ghana, Korle Bu, Accra, Ghana; 3 School of Public Health, University of Ghana, Legon, Accra, Ghana; 4 Research Directorate, Korle Bu Teaching Hospital, Korle Bu, Accra, Ghana

**Keywords:** Antibiotics, Antibiotic use, Surgical infections, Surgical antibiotic prophylaxis, Ghana

## Abstract

**Objective:**

The study aimed to assess antibiotic prescribing and use patterns at the Department of Surgery, Korle Bu Teaching Hospital.

**Design:**

A cross-sectional study design was employed in this study.

**Setting:**

The study was conducted at the Department of Surgery, Korle Bu Teaching Hospital.

**Participants:**

Forty-two prescribers out of 63 (67%) at the Department of Surgery responded to questionnaires. Over the study period, prescriptions and medical records of 1715 patients from the general surgery, neurosurgery, and urology units were reviewed.

**Main Outcome Measures:**

Percentage of prescriptions with antibiotics, percentage of prescribers using guidelines for antibiotic prescriptions, and percentage using culture and sensitivity to inform antibiotic prescriptions.

**Results:**

Of the 1715 prescriptions assessed, 75% (1294/1715) were from inpatients, and 45% (772/1715) included an antibiotic. Ciprofloxacin and metronidazole constituted 54% of antibiotic prescriptions from general surgery. Amoxicillin/clavulanic acid and ceftriaxone constituted 64.7% of antibiotic prescriptions from neurosurgery, and ceftriaxone and ciprofloxacin made up 37.7% of antibiotic prescriptions from urology. Microbiology testing was done for only 14.5% (9/62) of inpatients who received antibiotics for treatment. The choice of antibiotics was influenced mainly by doctors' previous experience (37/42, 88.1%).

**Conclusion:**

Antibiotics are widely used. About half of all prescriptions had antibiotics, with ciprofloxacin and metronidazole constituting more than half of antibiotic prescriptions from general surgery. Doctors mainly based their antibiotic prescriptions on previous experience and occasionally on microbiological investigations.

**Funding:**

None declared

## Introduction

Antibiotics are among the most commonly prescribed medications worldwide and are widely used to prevent and treat infectious diseases.^1^ They account for nearly 30-50% of all new and repeat prescriptions each year^2^ and contribute significantly to the cost of therapy.^3^, ^4^. The use of antibiotics has contributed significantly to the reduction of infection-associated mortality.^2^

Antibiotics are among the medications used frequently on surgical wards.^1^ Although antibiotics are extremely important in patient care, not all antibiotics can be justified.^1^,^2^ The problem with inappropriate use of antibiotics in hospitals, therefore, involves excessive use and underutilisation.^1^

In low- and middle-income countries (LMIC) like Ghana, relatively high levels of availability and consumption have led to disproportionately higher incidences of inappropriate use and greater resistance levels compared to high-income countries.^5^,^6^ In addition to increased risk of resistance, misuse of antibiotics can result in unnecessary exposure to medications, adverse events and increased costs and duration of hospitalisation for in-patients.^3^,^7^

Studies in Ghana have shown substantial bacterial resistance to commonly prescribed antibiotics,^8^ prolonged antibiotic treatment durations,^8^,^9^ frequent use of broad-spectrum agents,^9^ and a lack of awareness of factors contributing to antibiotic resistance. Though a policy document on antimicrobial use and resistance for Ghana 10 and a Ghana National Action Plan on Antimicrobial Resistance^11^ was launched in 2018 by the Ministry of Health, implementation is still in its teething stages. Ghana has a standard treatment guideline^12^, a regularly updated guideline that practitioners refer to for the prescription of drugs, including antibiotics. Some institutions have drug formularies for prescription by their prescribers that are peculiar to their needs.

The pattern of antibiotic use varies depending on the setting, prevalent strains of microorganisms, pattern of nosocomial infections, cost of antibiotics and medicines availability.^1^,^2^ Given this, the development of appropriate measures to monitor antibiotic use in health facilities is of utmost importance since medicines use in hospitals can considerably influence further medicine use outside hospitals.^6^,^13^ This study sought to evaluate the pattern of antibiotic prescribing and use at the Department of Surgery, Korle Bu Teaching Hospital (KBTH), and assess the choice of antibiotics. The study also sought to ascertain prescribers' views on antibiotic prescribing practices and identify prescribers' influences on antibiotic prescribing decisions.

## Methods

### Study design and setting

This was a hospital-based cross-sectional study conducted at the surgical outpatient department (OPD) and surgical in-patient wards at KBTH to assess the antibiotic prescribing and use pattern from 5th February to 7th May 2019. In addition, questionnaires were administered to prescribers in the department to assess their views on antibiotic prescribing practices. The Korle-bu teaching hospital is a 2000-bed capacity hospital with an average daily attendance of 1500 patients, about 250 of whom are admitted. The Department of Surgery is the largest in the hospital, comprising five sub-Budget Management Centres (BMC) (the Allied Health sub-BMC, Burns and Reconstructive Centre, Cardiothoracic Centre, Surgical sub-BMC, and the Trauma and Orthopaedics sub-BMC). A total of 81,833 cases are attended to annually in the surgical outpatient department, of which 8,749 are admitted. The surgical sub-BMC, with a 200-bed capacity, consists of general surgery and its sub-specialties, such as urology and neurosurgery. Each unit runs daily outpatient clinics from Monday to Friday. The department has a pharmacy unit in the surgical block near the outpatient clinic. The pharmacy unit has 16 staff, of which 10 pharmacists attend to both out and in-patients. It receives and serves an average of 27,427 prescriptions annually.^14^

### Sample size

The study included all prescription forms and medical records of patients cared for at OPD and as in-patients in units under the Department of Surgery over the study period.

### Inclusion and exclusion criteria

Prescriptions and medical records for all patients attended to at the units under study during the study period were included. Antibiotic prescriptions for patients for the purpose of treatment and surgical prophylaxis given within the recommended duration of 24 hours were included in the study. Antibiotic prescriptions for surgical prophylaxis given beyond the recommended 24 hours were also included in the study. For exclusion criteria, all house officers on all the wards under the study were excluded, as they had less than one year of experience in medical practice. House officers prescribe based on instructions from their supervisors and do not decide on antimicrobial prescribing independently.

### Data Collection

This involved extracting data from patients' prescriptions and medical records and administering questionnaires to prescribers at the surgical department.

### Antibiotic use in surgery

Research assistants were trained to support data extraction from prescription forms and data collection with the prescribers. A data collection sheet was designed and used to collect information on the prescription forms of patients. Data on patient demographics, medications prescribed, antibiotic characteristics, and antibiotic use at the department were extracted using the tool. To ensure that prescriptions of all patients treated at the surgical department were included, a pre-study sensitisation was done to inform prescribers to put down the diagnosis of patients on the prescription forms. Prescribers were also given special collection boxes in the consulting rooms where duplicates of prescriptions were kept. Research assistants picked up these duplicates daily throughout the study. Further, a second layer system was put in place at the surgical pharmacy to ensure that duplicates of prescriptions that were not detached and dropped in the box in the consulting room were detached and dropped in a box for daily pickups by the research assistants. Research assistants were also assigned to the wards within the surgical department to collect data on antibiotic prescriptions from the medical records of in-patients using data collection sheets. As part of data collection, patients for whom culture and sensitivity tests were requested and test results obtained were documented on the data collection sheets and organisms identified were documented.

### Perception of prescribers on antibiotic prescribing practices

All medical doctors who had practised at the Department of Surgery for a year or more were included in the study. A questionnaire was used to collect prescribers' views on antibiotic prescription. The questionnaire assessed prescribers' antibiotic selection criteria and procedures. Prescribers' views on the purpose of antibiotic use, the purpose of antibiotic prescribing, the request for culture and sensitivity testing, the influences on antibiotic prescribing decisions, and the guidelines used for antibiotic prescribing were sought.

### Data analysis

The data was coded and entered into Microsoft Excel 2016 spreadsheet and STATA version 15 for analysis. Descriptive statistics are presented in percentages and frequency tables. Continuous variables were summarised using mean with standard deviation or median with interquartile ranges and categorical data as percentages and frequencies. Antibiotic prescribing at the Department of Surgery for the various diagnoses of patients under study was compared with recommended antibiotic prescribing in the Ghana National Standard Treatment Guidelines. Descriptive statistics of the data gathered from prescribers were presented as frequencies and proportions.

### Ethics considerations

Ethical clearance for the study was obtained from the Ethics and Protocol Review Committee of Korle Bu Teaching Hospital (STC/IRB/00035/2018). Written informed consent was obtained from the doctors who responded to the questionnaires. Data extraction from prescription forms and medical records followed all ethical standards for using secondary and existing data for research. Anonymity and confidentiality were maintained at all times.

## Results

### Patient characteristics

A total of 1715 prescription forms and medical records were assessed, with 941 (54.9%) for male patients and 767 (44.7%) for female patients. Patient sex was not indicated on seven (0.4%) prescriptions and medical records (Table 1). The median age for prescriptions assessed was 51 years (Interquartile range (IQR) 36 to 64 years; range 4 months to 98 years). For in-patients, the median number of days of admission was 11 (IQR 5 to 22 days; range 1 to 96 days).

**Table 1:** Background information on prescriptions and characteristics of prescribers at the Department of Surgery

Prescriber characteristics	
Variable	Number (%)
**Sex indicated on records.**	
**Male**	941 (54.90)
**Female**	767 (44.70)
**Not indicated**	7 (0.40)
	
**Age indicated on records.**	
Median (IQR*) age	51 years (36 – 64) years
**Patient duration of hospital stay on record.**	
**Median (IQR) patient duration of hospital stay**	11 (5 – 22) days
	
**Characteristics of Prescribers**	
**Sex**	
**Male**	37 (88.10)
**Female**	5 (11.90)
**Rank**	
**Junior Resident**	24 (54.14)
**Specialist**	7 (16.67)
**Senior Specialist**	7 (16.67)
**Consultant**	4 (9.52)
	
**Years of practice**	
**Median (IQR), years**	5 (2 – 8)

* IQR - Interquartile range

Forty-two prescribers out of 63 (67%) responded to the questionnaires. Thirty-seven of the prescribers (88.1%) were males, and 5 (11.9%) were females (Table 1). Most prescribers were junior residents (24/42 (57.1%). The rest were specialists/senior residents (7/42 (16.67%)) and senior specialists/consultants (11/42 (26.19%)). The median years of practice of the prescribers was 5 years (IQR 2 to 8 years; range 1 to 25 years).

### Pattern of Antibiotic Prescription

The frequency of antibiotic prescription over the study period was 1291, constituting 25 different antibiotics. A total of 24 different antibiotics were prescribed at the general surgery unit, while at both the neurosurgery and urology units, 14 different antibiotics were prescribed throughout the study. Out of the total number of prescriptions and medical records assessed (1715), 243 (14.2%) were from the wards, while 1472 (85.8%) were from the surgical pharmacy and consulting rooms. Median number of medications per prescription was 2 (IQR 1-4; range 1-20). Antibiotics were prescribed on 772 (45%) of the 1715 prescriptions assessed, with a median number of antibiotics per prescription being 1 (IQR 1-2; range 0-6). Out of the 772 prescription forms and records with antibiotics prescribed, 24.6% (n=190) were for patients who were on admission to the wards, and 75.4% (n=582) were from the surgical pharmacy and consulting rooms.

Again, 48.3% (n=373/772) of the prescription forms and records with antibiotics prescribed were from the general surgery unit, 16.5% (n=127/772) from the neurosurgery unit, and 18.6% (n=144/772) from the urology unit. Prescription forms with no address indicated made up 16.8% (n=128/772) of the prescription forms and records with an antibiotic prescribed.

Further, the results indicate that the most frequently prescribed therapeutic groups included quinolones (24.24%), penicillins (20.60%), and nitroimidazoles (18.67%), while the least prescribed were tetracyclines (0.39%) and quinolone/nitroimidazole combination (0.15%) (Table 3).

**Table 3:** Therapeutic classes of antibiotics prescribed

Therapeutic Class (Total, *%)	Antibiotic	Number (**%)
**Quinolone (313, 24.2%)**	Ciprofloxacin	234 (74.8%)
	Levofloxacin	79 (25.2%)
**Penicillins (266, 20.6%)**	Amoxicillin/Clavulanic Acid	240 (90.2%)
	Amoxicillin	11 (4.1%)
	Flucloxacillin	12 (4.5%)
	Benzylpenicillin	2 (0.8%)
	Phenoxymethylpenicillin	1 (0.4%)
**Nitroimidazole (241, 18.7%)**	Metronidazole	241(100.0%)
**Cephalosporins (211, 16.3%)**	Cefuroxime	53 (25.1%)
	Ceftriaxone	151 (71.6%)
	Cefpodoxime	4 (1.9%)
	Cefixime	2 (0.9%)
	Ceftazidime	1 (0.5%)
**Lincosamide (88, 6.8%)**	Clindamycin	88 (100.0%)
**Aminoglycosides (63, 4.9%)**	Amikacin	3 (4.8%)
	Gentamycin	60 (95.2%)
**Carbapenem (37, 2.9%)**	Meropenem	37 (100.0%)
**Macrolides (27, 2.1%)**	Azithromycin	19 (70.4%)
	Clarithromycin	7 (25.9%)
	Erythromycin	1 (3.7%)
**Glycopeptides (20, 1.6%)**	Vancomycin	20 (100.0%)
**Nitrofuran (18, 1.4%)**	Nitrofurantoin	18 (100.0%)
**Tetracycline (5, 0.4%)**	Doxycycline	4 (80.0%)
	Tetracycline	1 (20.0%)
**Quinolone/Nitroimidazole (2, 0.2%)**	Ciprofloxacin/Tinidazole	2 (100.0%)

* *% - percentage of therapeutic class of total (N=1291)*

** *% - percentage of antibiotics of a therapeutic class*

### Influences and guidelines for antibiotic prescribing

**Figure 1:**
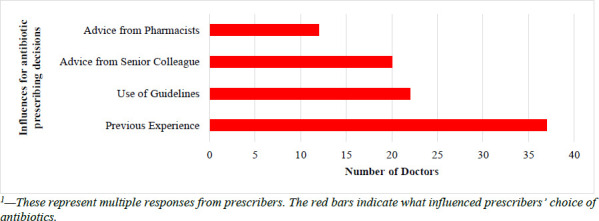
Influences on the prescribing decisions of prescribers for antibiotics

All the prescribers who answered the questionnaires confirmed that they prescribed antibiotics for prophylaxis or treatment. Each prescriber indicated at least one reason that influenced their choice of antibiotics. Most of the prescribers indicated that the choice of antibiotics prescribed was based on their previous experiences (88.1%; n=37). Other influences on antibiotic prescribing included the use of guidelines (52.4%; n=22), advice from senior colleagues (47.6%; n=20), and advice from pharmacists (28.6%; n=12) (Figure 1). Guidelines often used by doctors included the Ghana Standard Treatment Guideline (STG) (n=16) and the British National Formulary (BNF) (n=12).

### Purpose of antibiotic prescription

**Figure 2:**
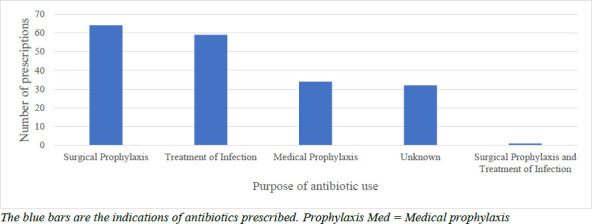
Purpose of antibiotic prescription at the Department of Surgery

Of the total number of prescriptions assessed, 1715, 243 (14.2%) were from the wards, while 1472 (85.8%) were from the surgical pharmacy and consulting rooms. Antibiotics were prescribed on a total of 190 (77%) of the 243 prescriptions assessed from the wards. Out of the total 190 prescriptions forms and records that had antibiotics prescribed on the ward, indications for antibiotic therapy for prescriptions from the wards included surgical prophylaxis (n=64; 33.7%), treatment of infections (n=59; 31.1%), medical prophylaxis (patients with surgical conditions who were given antibiotic prophylaxis for chronic medical conditions) (n=34; 17.9%) and both surgical prophylaxis and treatment of infection (0.5%; n=1). The indication for which antibiotics were prescribed for 16.8% (n=32) of the patients could not be determined based on the diagnoses indicated in their medical records (Figure 2).

### Microbiology

Culture and sensitivity tests were done for 14.5% of 9 of the 62 inpatients who received antibiotics for treatment. Samples taken included urine (55.6%; (5/9)), wound swabs (33.3%; (3/9)) and pus (11.1%; (1/9)). Organisms isolated included *Escherichia coli* (60%; (6/10)), *Pseudomonas aeruginosa* (10%; (1/10)) and *Staphylococcus saprophyticus* (10%; (1/10)). No bacterial growth was observed in two samples.

### Diagnoses and antibiotic prescriptions

The most common antibiotic combination prescribed for treatment was Ciprofloxacin with metronidazole (n=10, 16.7%). Details of various antibiotics prescribed and the diagnosis for the prescription during their hospital stay are shown in Appendix A.

## Discussion

The study, which evaluated the pattern of antibiotic prescribing and use at the department of surgery of a large teaching hospital in a sub-Saharan African country, showed that antibiotics are prescribed largely with no microbiology test backing the prescriptions. The study also sought to ascertain prescribers' views on antibiotic prescribing practices, and it was found that the choice of antibiotics was mainly based on doctors' previous experience. This has public health implications in the era of antimicrobial resistance.^2^

The study further found that most patients receive antibiotics at some point during their hospitalisation. This is consistent with other studies that estimate that 80% of adult and paediatric patients receive antibiotics during their hospitalisation, with the majority being inappropriate or unnecessary.^15^,^16^,^17^

Practitioners believe patients are unwell if they require admission and tend to preemptively start them on antibiotics before a final diagnosis is made. Surgical antibiotics prophylaxis must be clearly distinguished from pre-emptive use of antibiotics to treat early infection, which may not be clinically apparent.^18^,^19^,^20^ Education on the judicious use of antibiotics in the hospital environment is essential.^2^ Appropriate antibiotic prophylaxis can help reduce the risk of postoperative infections. Still, prolonged antibiotic use for prophylaxis, as found in this study, increases the selective pressure supporting the emergence of antimicrobial resistance. A survey on antibiotic prescribing practices in general surgery also revealed similar findings where patients received antibiotics for prolonged periods beyond the single-dose prophylaxis.^13^

This study shows several antibiotic combination therapies prescribed for patients, the commonest being ciprofloxacin with metronidazole and ciprofloxacin with clindamycin. The findings from this study were similar to those of a survey conducted on antibiotic use among hospitalised patients in northern Nigeria.^21^ The frequently used antibiotics could be because most prescriptions were from the general surgery unit where the usual gram-negative bacteria and anaerobic organisms associated with abdominal infections and procedures are pre-dominant.^21^,^22^ Due to patient economic challenges and the absence of adequate diagnostic infrastructure, antibiotics may often be prescribed empirically as combination therapies in our setting since prescribers would want to provide coverage for all likely pathogens.^21^ This was evident in this study. The different antibiotic combination therapies for similar conditions in this study bring to the fore the need for institutional-based antimicrobial guidelines and institutional-based antimicrobial stewardship programs.^21^

Enhancing the prudent use of antibiotics and attaining substantial improvements in antibiotic use requires a full understanding of healthcare professionals' attitudes toward antibiotic prescribing and assessing their knowledge about the growing problem of resistance.^23^ The study demonstrated that the availability of guidelines does not seem to influence the decision to prescribe antibiotics. Prescribers continue with old prescribing methods as passed down from generations. This could be due to the absence of hospital or departmental-based antimicrobial prescribing guidelines or formularies.^23^,^24^ This may also be due to the non-adherence of healthcare providers to established clinical practice guidelines for the management of common infections for fear of not adequately preventing or treating infections. 23,24

Development of institutional or departmental-based antimicrobial prescribing guidelines and antibiograms, which are more tailored towards the pattern of infections in the setting, may be more beneficial in optimising antibiotic prescribing than depending on national guidelines.^24^ Enhancement of the numbers and roles of clinical pharmacists can also positively influence antibiotic prescribing and choice, especially in settings where prescribers prefer consulting senior colleagues to engaging with clinical pharmacists in antibiotic use decisions due to the unavailability of Pharmacists on the wards.

### Limitation

The current study's findings should be interpreted in the context of some limitations. Not all prescriptions were accessible for analysis in this study since patients registered with the National Health Insurance Scheme (NHIS) presented their prescriptions at NHIS-accredited Pharmacies outside the hospital. Some duplicates were not detached from prescriptions written in the consulting rooms by the doctors and nurses, resulting in fewer prescription duplicates being collected from the consulting rooms for this study. However, the sample of 1715 used for analysis and interpretation in this study is adequate to justify the study's conclusions. We are limited in generalising these findings to the entire population. Only 42 of the 63 prescribers responded to questionnaires. Hence, we are unable to generalise the findings to the entire population. No inferential statistics were made on the data acquired here. Pre-study sensitisation of prescribers may have influenced their prescription patterns during the study period.

## Conclusion

Antibiotics are widely used in the surgical department of the Korle-Bu Teaching Hospital in Ghana. About half of all prescriptions contain antibiotics, with ciprofloxacin and metronidazole constituting more than half of antibiotic prescriptions from general surgery. Doctors mainly base their prescriptions on previous experience and the advice of senior colleagues and occasionally rely on microbiological investigations.

The current situation regarding antibiotic prescriptions in the Korle-Bu Teaching Hospital surgical unit is fertile ground for microbial resistance. This underscores the need for further research to understand antibiotic prescribing behaviour better and incorporate effective antibiotic stewardship strategies with input from local experts.

## Figures and Tables

**Table 2:** Pattern of Antibiotic Prescribing at the Department of Surgery

Variable	Frequency (%)	Median (IQR*)
**Location of medication prescription**		
**Ward**	243 (14.2%)	
**Surgical pharmacy and consulting rooms**	1472 (85.8%)	
		
**Types of prescriptions**		
**Prescription with antibiotic**	772 (45%)	
**Prescription with no antibiotic**	943 (55%)	
		
**Number of medications per prescription**	2	1-4 (range 1-20)
**Number of antibiotics per prescription**	1	1-2 (range 0-6)
		
**Source of antibiotic prescription**		
**From the ward**	190 (24.6%)	
**Surgical pharmacy and consulting rooms**	582 (75.4%)	
**Units where antibiotics were prescribed from**		
**General Surgery**	373/772 (48.3%)	
**Neurosurgery**	127/772 (16.5%)	
**Urology**	144/772 (18.6%)	
**No unit indicated**	128/772 (16.8%)	
**Number of different antibiotics prescribed per unit**		
**General Surgery**	24 (46.2%)	
**Neurosurgery**	14 (26.9%)	
**Urology**	14 (26.9%)	

* IQR – Interquartile range

## Appendix

**Appendix A:** Antibiotic prescriptions and corresponding diagnoses

Antibiotic combination (n, %)	Patient diagnoses	Number of patients
**Amoxicillin/Clavulanic acid (3, 5.0)**	Cellulitis	1
	Cholecystitis	1
	Right Inguinal Ulcer	1
**Amoxicillin/Clavulanic acid + Ceftriaxone (1, 1.67)**	Guillain-Barre Syndrome, Transverse Myelitis	1
		
**Amoxicillin/Clavulanic acid + Ceftriaxone + Azithromycin (1, 1.67)**	**Leg / Foot Gangrene**	**1**
**Amoxicillin/Clavulanic acid + Ciprofloxacin + Clindamycin (1, 1.67)**	Cellulitis	1
**Amoxicillin/Clavulanic acid + Clindamycin (1, 1.67)**	Wound Infection	1
**Amoxicillin/Clavulanic acid + Metronidazole (5, 8.33)**	Acute Pancreatitis	1
	Leg / Foot Gangrene	3
	Urinary Tract Infection	1
**Amoxicillin/Clavulanic acid + Metronidazole + Ceftriaxone (2, 3.33)**	Lumbar Canal Stenosis	1
	Subdural Empyema	1
**Amoxicillin/Clavulanic acid + Metronidazole + Ceftriaxone + Amikacin, (1, 1.67)**	Above Knee Amputation	1
**Amoxicillin/Clavulanic acid + Nitrofurantoin + Ceftriaxone (1, 1.67)**	Fournier Gangrene, Sepsis	1
**Azithromycin + Metronidazole+ Ciprofloxacin + Clindamycin + Amikacin, (1, 1.67)**	Leg / Foot Gangrene	1
		
**Cefuroxime (2, 3.33)**	**Acute Appendicitis**	**1**
	Cholecystitis	1
**Ceftriaxone (1, 1.67)**	Epidydymo-Orchitis, Testicular Torsion	1
**Ceftriaxone + Ciprofloxacin + Metronidazole (1, 1.67)**	Sepsis	1
**Ceftriaxone + Clindamycin (1, 1.67)**	Cellulitis, Sepsis	1
**Ceftriaxone + Clindamycin + Amoxicillin/Clavulanic acid + Metronidazole + Gentamicin (1, 1.67)**	Subdural Empyema	1
		
**Ceftriaxone + Gentamicin + Clindamycin + Levofloxacin (1, 1.67)**	**Benign Prostate Hyperplasia**	**1**
**Ceftriaxone + Levofloxacin + Gentamicin (1, 1.67)**	Benign Prostate Hyperplasia	1
**Ceftriaxone + Metronidazole + Azithromycin + Amoxicillin/Clavulanic acid (1, 1.67)**	Penetrating Chest and Abdominal Injury	1
**Ciprofloxacin (1, 1.67)**	Metastatic Rectal Cancer	1
**Ciprofloxacin + Azithromycin + Metronidazole + Ceftriaxone (1, 1.67)**	Intraabdominal infection from leaking bowel anastomoses	1
**Ciprofloxacin + Clindamycin (8, 13.33)**	Cellulitis	1
	Gangrene, Superimposed Ulcer	1
	Leg / Foot Gangrene	3
	Pyomyositis Of Right Thigh	1
	Right Chronic Leg Ulcer	1
	Sepsis	1
**Ciprofloxacin + Clindamycin + Metronidazole (1, 1.67)**	Cellulitis	1
**Ciprofloxacin + Doxycycline, + Clindamycin (1, 1.67)**	Scrotal Abscess	1
**Ciprofloxacin + Metronidazole+ Ceftriaxone + Cefixime + Ceftazidime (1, 1.67)**	Acute Appendicitis	1
**Ciprofloxacin + Metronidazole + Doxycycline, + Gentamicin (1, 1.67)**	Appendix Mass	1
**Ciprofloxacin + Metronidazole + Gentamicin (1, 1.67)**	Perforated duodenal ulcer	1
**Ciprofloxacin + Metronidazole + Levofloxacin + Gentamicin (1, 1.67)**	Peritonitis	1
**Clindamycin (1, 1.67)**	Sepsis	1
**Flucloxacillin + Metronidazole + Amoxicillin/Clavulanic acid (1, 1.67)**	Cellulitis	1
**Levofloxacin (2, 3.33)**	Sepsis	2
**Levofloxacin + Gentamicin (1, 1.67)**	Sigmoid Colon Perforation	1
**Meropenem (2, 3.33)**	Pyelonephritis	1
	Sepsis	1
**Metronidazole + Ciprofloxacin (10, 16.67)**	Acute Appendicitis	7
	Gangrenous bowel	1
	Intestinal bowel perforation	1
	Perforated duodenal ulcer	1
**Phenoxymethylpenicillin + Gentamicin + Clindamycin (1, 1.67)**	Cellulitis	1
